# The Proinflammatory Cytokine High-Mobility Group Box-1 Mediates Retinal Neuropathy Induced by Diabetes

**DOI:** 10.1155/2014/746415

**Published:** 2014-03-10

**Authors:** Ahmed M. Abu El-Asrar, Mohammad Mairaj Siddiquei, Mohd Imtiaz Nawaz, Karel Geboes, Ghulam Mohammad

**Affiliations:** ^1^Department of Ophthalmology, College of Medicine, King Saud University, Riyadh, Saudi Arabia; ^2^Department of Ophthalmology, King Abdulaziz University Hospital, Old Airport Road, P.O. Box 245, Riyadh 11411, Saudi Arabia; ^3^Laboratory of Histochemistry and Cytochemistry, University of Leuven, Leuven, Belgium

## Abstract

To test the hypothesis that increased expression of proinflammatory cytokine high-mobility group box-1 (HMGB1) in epiretinal membranes and vitreous fluid from patients with proliferative diabetic retinopathy and in retinas of diabetic rats plays a pathogenetic role in mediating diabetes-induced retinal neuropathy. Retinas of 1-month diabetic rats and HMGB1 intravitreally injected normal rats were studied using Western blot analysis, RT-PCR and glutamate assay. In addition, we studied the effect of the HMGB1 inhibitor glycyrrhizin on diabetes-induced biochemical changes in the retina. Diabetes and intravitreal injection of HMGB1 in normal rats induced significant upregulation of HMGB1 protein and mRNA, activated extracellular signal-regulated kinase 1 and 2 (ERK1/2), cleaved caspase-3 and glutamate; and significant downregulation of synaptophysin, tyrosine hydroxylase, glutamine synthetase, and glyoxalase 1. Constant glycyrrhizin intake from the onset of diabetes did not affect the metabolic status of the diabetic rats, but it significantly attenuated diabetes-induced upregulation of HMGB1 protein and mRNA, activated ERK1/2, cleaved caspase-3, and glutamate. In the glycyrrhizin-fed diabetic rats, the decrease in synaptophysin, tyrosine hydroxylase, and glyoxalase 1 caused by diabetes was significantly attenuated. These findings suggest that early retinal neuropathy of diabetes involves upregulated expression of HMGB1 and can be ameliorated by inhibition of HMGB1.

## 1. Introduction

Diabetic retinopathy (DR), a vision-threatening disease, has classically been regarded as a disease of the retinal microvasculature and a consequence of vascular cell damage. However, recent studies proved that neurodegeneration and impaired visual function are initiated early after the onset of diabetes and progress independently of the vascular lesions [1–4]. However, the molecular mechanisms underlying the diabetes-induced retinal neurodegeneration and dysfunction are still not well understood.

High-mobility group box-1 (HMGB1) is a nonhistone DNA-binding nuclear protein that has been implicated in diverse intracellular functions, including the stabilization of nucleosomal structures and the facilitation of gene transcription. Necrotic cell death can result in passive leakage of HMGB1 from the cell as the protein is then no longer bound to DNA. In addition, HMGB1 can be actively secreted by different cell types, including activated monocytes and macrophages, mature dendritic cells, natural killer cells, and endothelial cells. Extracellular HMGB1 functions as a proinflammatory cytokine and triggers the inflammatory response through the activation of multiple receptors such as the receptor for advanced glycation end products (RAGE), toll-like receptor-2 (TLR2), and TLR4 leading to activation of the transcription factors extracellular signal-regulated kinase 1 and 2 (ERK1/2) and nuclear factor Kappa B (NF-*κ*B), which may alter gene transcription and lead to the upregulation of proinflammatory cytokines, chemokines, and adhesion molecules and intensifies cellular oxidative stress [5–10], processes that may play a role in the pathogenesis of diabetic retinal neurodegeneration and dysfunction. Strong evidence indicates that chronic, low-grade inflammation is implicated in the pathogenesis of DR [11, 12]. Recently, it was demonstrated that HMGB1 is the main mediator bridging persistent neuroinflammation and chronic progressive dopaminergic neurodegeneration in neurodegenerative diseases, such as Parkinson's disease [13].

Recently, HMGB1 has received particular attention with respect to its pathological role in cerebral ischemia. After ischemic injury induced by transient middle cerebral artery occlusion in mice and rats, HMGB1 was found to be translocated into the cytoplasmic compartment from nuclei and released into the extracellular space from neurons [14–18]. In these studies, extracellular HMGB1 plays a key role in the development of neuronal injury through microglial activation, induction of apoptosis, excitatory amino acid release, and induction of proinflammatory mediators [14–19]. Downregulation of HMGB1 or treatment with neutralizing anti-HMGB1 monoclonal antibody remarkably suppressed infarct size, activation of microglia, and induction of proinflammatory markers and inhibited the increased permeability of the blood-brain barrier [14, 18].

In previous studies, we demonstrated that HMGB1 and RAGE were expressed by vascular endothelial cells and stromal cells in fibrovascular epiretinal membranes from patients with proliferative diabetic retinopathy (PDR). In addition, we demonstrated increased levels of HMGB1 in the vitreous samples from patients with PDR and that there were significant positive correlations between the vitreous levels of HMGB1 and the levels of the inflammatory biomarkers [20–22]. Furthermore, we demonstrated that diabetes induced significant upregulation of the expression of HMGB1 and RAGE in the retinas of rats and mice and that intravitreal administration of HMGB1 to normal rats induced activation of inflammatory signaling pathways in the retina and increased retinal vascular permeability [21, 23].

Glycyrrhizin (GA), an ingredient of the licorice roots, has long been known to exhibit glucocorticoid-like anti-inflammatory actions by inhibiting 11*β*-hydroxysteroid dehydrogenase. GA has been shown to have anti-inflammatory and antiviral effects. More recently, GA has also been shown to bind to and inhibit chemoattractant, mitogenic, and cytokine-like activities of HMGB1 [24]. In this study, we explored the hypothesis that HMGB1 plays a pathogenetic role in mediating diabetes-induced retinal neuropathy. To test this hypothesis, we investigated the expression of the neurodegeneration mediators and markers cleaved caspase-3, synaptophysin, tyrosine hydroxylase (TH), glutamine synthetase (GS), glutamate, and glyoxalase 1 (GLO 1) in the retinas of diabetic rats and in the retinas of normal rats after intravitreal administration of HMGB1. In addition, we analyzed whether constant GA intake suppresses retinal neuropathy induced by diabetes in rats.

## 2. Materials and Methods

### 2.1. Animals 

#### 2.1.1. Induction of Diabetes and Glycyrrhizin Treatment

All procedures with animals were performed in accordance with the Association for Research in Vision and Ophthalmology (ARVO) statement for use of animals in ophthalmic and vision research and were approved by the institutional animal care and use committee of the College of Pharmacy, King Saud University. Adult male Sprague Dawley rats of 8-9 weeks of age (200−220 g) were overnight fasted and streptozotocin (STZ) 55 mg/kg in 10 mM sodium citrate buffer, pH 4.5 (Sigma, St. Louis, MO), was injected intraperitoneally. Equal volumes of citrate buffer were injected in nondiabetic animals. Rats were considered diabetic if their blood glucose was greater than 250 mg/dL. Age-matched normal rats served as controls.

Diabetic rats were divided into 2 groups: the rats in group I received normal drinking water without any supplementation, and group II received drinking water supplemented with glycyrrhizic acid (150 mg/kg/day, Santa Cruz Biotechnology, Inc., Santa Cruz, CA) immediately after establishment of diabetes throughout the course of the experiment. Each group had 8–12 rats. After 4 weeks of diabetes, the rats were euthanized by an overdose of chloral hydrate, the eyes were removed, and retina was isolated and frozen immediately in liquid nitrogen and stored at −80°C to be analyzed by Western blot analysis or biochemical assay.

#### 2.1.2. Intravitreal Injection of HMGB1

Sprague Dawley rats (200−220 g) were kept under deep anesthesia, and sterilized solution of recombinant HMGB1 (5 ng/5 *μ*L; R&D Systems, Minneapolis, MN) was injected into the vitreous of the right eye as previously described by us [23]. For the control, the left eye received 5 *μ*L of sterile phosphate buffer saline (PBS). The animals were sacrificed 4 days after intravitreal administration, and the retinas were carefully dissected, snap-frozen in liquid nitrogen, and stored at −80°C to be analyzed by Western blot analysis or biochemical assay. The breakdown of blood-retina barrier induced by intravitreal injection of HMGB1 might lead to raised levels of HMGB1 in the serum. Therefore, we determined the levels of HMGB1 in equal amounts of serum from intravitreally injected rats and normal rats using Western blot analysis.

#### 2.1.3. Western Blot Analysis

Retinas were homogenized in a western lysis buffer (30 mM Tris-HCl; pH 7.4, 250 mM Na_3_VO_4_, 5 mM EDTA, 250 mM sucrose, 1% Triton X-100 with Protease inhibitor). The lysate was centrifuged at 14,000 ×g for 10 min at 4°C, and the supernatant was collected. Protein content was assayed by DC protein assay (Bio-Rad Laboratories, Hercules, CA). The tissue lysate containing 40–50 *μ*g of protein was separated on 10% or 12% SDS-polyacrylamide gels and was transferred onto nitrocellulose membranes. The blots were blocked with 5% nonfat milk in TBST (20 mM Tris-HCl, pH 7.6, 136 mM NaCl, and 0.1% Tween-20).

For detection of HMGB1, phospho-ERK1/2, synaptophysin, cleaved caspase-3, TH, GLO1, and GS, the membrane was incubated overnight at 4°C with rabbit polyclonal anti-HMGB1 (1 : 1000, Cat. number ab18256, Abcam, UK), rabbit monoclonal anti-phospho-ERK1/2 (0.5 *μ*g/mL, Cat. number MAB1018, R&D Systems), mouse monoclonal anti-ERK1/2 (0.5 *μ*g/mL, Cat. number MAB1576, R&D Systems), goat polyclonal anti-synaptophysin (1 *μ*g/mL, Cat. number AF-5555, R&D Systems), rabbit polyclonal anti-cleaved caspase-3 (1 : 300, Cat. number SC-7148, Santa Cruz), mouse monoclonal anti-TH (0.5 *μ*g/mL, Cat. number MAB7566, R&D Systems), rabbit polyclonal anti-GLO1 (1 : 200, Cat. number ab96032, Abcam), and goat polyclonal anti-GS (1 : 500, sc-6640, Santa Cruz). After overnight incubation with primary antibodies, the membranes were washed four times with TBS-T (5 min each). For synaptophysin and GS, the membrane was incubated at room temperature for 1.5 h with anti-goat secondary horseradish peroxidase-conjugated antibody (1 : 2000, SC-2768, Santa Cruz), for HMGB1, phospo-ERK1/2 cleaved caspase-3 and GLO1, with anti-rabbit secondary horseradish peroxidase-conjugated antibody (1 : 2000, SC-2004, Santa Cruz), and for ERK1/2 and TH with anti-mouse secondary horseradish peroxidase-conjugated antibody (1 : 2000, SC-2005, Santa Cruz). After incubations with secondary antibodies, membranes were washed four times with TBS-T (5 min each) and the immunoreactivity of bands was visualized on a high-performance chemiluminescence machine (G: Box Chemi-XX8 from Syngene, Synoptic Ltd. Cambridge, UK) by using enhanced chemiluminescence plus Luminol (sc-2048, Santa Cruz) and quantified by densitometric analysis using image processing and analysis in GeneTools (Syngene by Synoptic Ltd. Cambridge, UK). For loading control, the blots were stripped and detected with a mouse monoclonal anti-*β*-actin (1 : 2000, SC-2048, Santa Cruz) antibody. For phosphor-ERK1/2, the loading control was total ERK1/2. All data from the three independent experiments were expressed as a ratio to optical density.

#### 2.1.4. Real-Time Reverse Transcription Polymerase Chain Reaction (RT-PCR)

Total RNA was extracted from retina using TRI reagent (Ambion, TX), according to manufacturer's protocol. cDNA was synthesized from 1 *μ*g RNA, using a high capacity cDNA reverse transcription kit (Applied Biosystem, CA) following manufacturer's instruction. RT-PCR was performed using a SYBR green PCR master mix. The PCR primers for rat were HMGB1 forward 5′-TGATTAATGAATGAGTTCGGGC-3′ reverse 5′- TGCTCAGGAAACTTGACTGTTT-3′ and *β*-actin forward 5′-CCTCTATGCCAACACAGTGC-3′ reverse 5′-CATCGTACTCCTGCTTGCTG-3′. The standard PCR conditions included 2 minutes at 50°C and 10 min at 95°C followed by 40 cycles of extension at 95°C for 15 seconds and one minute at 60°C. Threshold lines were automatically adjusted to intersect amplification lines in the linear portion of the amplification curves and cycle to threshold (Ct) was recorded automatically. Data were normalized with *β*-actin mRNA level (housekeeping gene) and the fold change in gene expression relative to normal was calculated using the ddCt method.

#### 2.1.5. Measurement of Glutamate in Rat Retinas

Glutamate level was measured in retinal homogenate by using glutamate assay kit (Cat. number ab83389, Abcam), according to the manufacturer's instruction. Briefly, 50 *μ*L of standards and retinal homogenate (equal amount) was loaded in a clear 96-well plate, followed by addition of 100 *μ*L reaction mix solution having assay buffer, glutamate developer, and glutamate enzyme mix. The plate was incubated for 30 minutes at 37°C (protected from light) and was read at 450 nm in microplate reader (Stat Fax 4200 microplate reader, awareness technology, Palm City, FL). The concentration of measured glutamate was expressed as nmol/*μ*L/*μ*g of protein.

#### 2.1.6. Statistical Analysis

The Mann-Whitney test was used to compare means from two independent groups. A *P* value less than 0.05 indicated statistical significance. SPSS version 12.0 was used for the statistical analyses.

## 3. Results

### 3.1. Severity of Hyperglycemia in Rats

The body weights of the diabetic rats were lower and their blood glucose levels were more than fourfold higher compared with age-matched normal control rats (178 ± 22 versus 287 ± 28 g and 475 ± 32 versus 111 ± 12 mg/dL, resp.). Treatment of the diabetic rats with GA for one month did not change these metabolic variables in the diabetic rats (167 ± 25 versus 178 ± 22 g and 449 ± 36 versus 475 ± 32 mg/dL, resp.).

### 3.2. Effect of Diabetes on Retinal Expression of Mediators and Markers of Neurodegeneration

**Figure 1:**
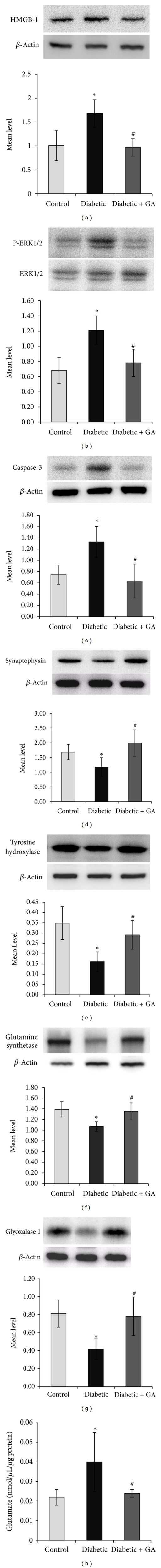
Western blot analysis of high-mobility group box-1 (HMGB1), extracellular signal-regulated kinase 1 and 2 (ERK1/2), cleaved caspase-3, synaptophysin, tyrosine hydroxylase, glutamine synthetase, and glyoxalase 1 in rat retinas. *β*-Actin was used as a housekeeping control. Protein expression of ERK1/2 activation (phosphorylation) was quantified by Western blot analysis using phospho- (P-) ERK1/2 specific antibody and was adjusted to the protein levels of unphosphorylated ERK1/2 antibody in each sample. There is a significant increase in the expression of HMGB1 (a), activated ERK1/2 (b), and cleaved caspase-3 (c) and a significant decrease in the expression of synaptophysin (d), tyrosine hydroxylase (e), glutamine synthetase (f), and glyoxalase 1 (g) in the retinas of diabetic rats compared to nondiabetic controls. Glycyrrhizic acid (GA) significantly attenuated diabetes-induced upregulation of HMGB1 (a), activated ERK1/2 (b), and cleaved caspase-3 (c) and diabetes-induced downregulation of synaptophysin (d), tyrosine hydroxylase (e), GS (f), and glyoxalase 1 (g). Glutamate assay revealed a significant increase in glutamate levels in the retinas of diabetic rats compared to nondiabetic controls. The levels of glutamate in the GA-treated diabetic rats were significantly less than those in the untreated diabetic rats (h). Each experiment was repeated at least 3 times with fresh samples. A representative set of samples is shown. Results are expressed as mean ± SD of at least 6 rats in each group. *P < 0.05 compared with nondiabetic control rats. ^#^ *P* < 0.05 compared with diabetic rats.

Western blot analysis demonstrated significant upregulation of HMGB1 expression in diabetic retinas compared to nondiabetic retinas. The expression of HMGB1 protein in the retinas of diabetic rats was upregulated by about 66% as compared to the retinas of nondiabetic rats (Figure 1(a)). Diabetes significantly increased ERK1/2 activation in the retinas by about 77% compared to nondiabetic controls (Figure 1(b)). Cleaved caspase-3, the apoptosis executer enzyme, was significantly upregulated in diabetic retinas compared to nondiabetic controls. Cleaved caspase-3 levels in the retinas of diabetic rats were increased by about 70% compared to nondiabetic controls (Figure 1(c)). The synaptic vesicle protein synaptophysin and the dopaminergic amacrine cell marker TH levels obtained in diabetic animals were significantly lower than those of nondiabetic animals. The levels decreased by about 68% and 46%, respectively (Figures 1(d) and 1(e)). GS, an enzyme that converts glutamate into glutamine, protein expression was significantly decreased in diabetic retinas compared to nondiabetic retinas. GS levels decreased by about 75% (Figure 1(f)). GLO1, an enzyme critical for the detoxification of advanced glycation end products (AGEs), protein expression was significantly decreased in diabetic retinas compared to nondiabetic rats. GLO1 expression in diabetic retinas decreased by about 51% (Figure 1(g)). Glutamate assay revealed that glutamate levels in the retinas of diabetic animals (0.04 ± 0.015 nmol/*μ*L/*μ*g protein) were significantly higher than those in nondiabetic controls (0.022 ± 0.004 nmol/*μ*L/*μ*g protein) (*P* = 0.036) (Figure 1(h)).

### 3.3. Effect of Intravitreal Administration of HMGB1 on Retinal Expression of Mediators and Markers of Neurodegeneration in Normal Rats

**Figure 2:**
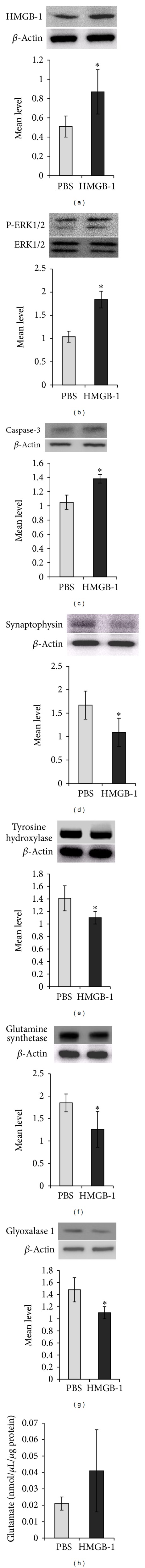
Western blot analysis of rat retinas. Intravitreal administration of high-mobility group box-1 (HMGB1) induced a significant upregulation of the expression of HMGB1 (a), activated extracellular signal-regulated kinase 1 and 2 (ERK1/2) (b), and cleaved caspase-3 (c) and a significant downregulation of the expression of synaptophysin (d), tyrosine hydroxylase (e), glutamine synthetase (f), and glyoxalase 1 (g) compared to intravitreal administration of phosphate buffer saline (PBS). Glutamate assay revealed that injection of HMGB1 tended to increase retinal glutamate expression, but this was not significant (h). Each experiment was repeated at least 3 times with fresh samples. A representative set of samples is shown. Results are expressed as mean ± SD of at least 6 rats in each group. *P < 0.05 compared with PBS.

There was no significant difference in serum levels of HMGB1 between rats intravitreally injected with HMGB1 (135.64 ± 14.38) and normal rats (134.88 ± 11.01) (*P* = 0.225). Intravitreal injection of HMGB1 resulted in increased HMGB1 expression by about 72% compared to the values obtained from the contralateral eye that received PBS alone (Figure 2(a)). In the same retinal samples, HMGB1 injection resulted in a 76% increase in ERK1/2 activation (Figure 2(b)), 31% increase in cleaved caspase-3 expression (Figure 2(c)), 65% decrease in synaptophysin expression (Figure 2(d)), 65% decrease in TH expression (Figure 2(e)), 70% decrease in GS expression (Figure 2(f)), and 71% decrease in GLO1 expression (Figure 2(g)). Injection of HMGB1 tended to increase retinal glutamate expression, but this was not significant (0.041 ± 0.025 versus 0.021 ± 0.004 nmol/*μ*L/*μ*g protein; *P* = 0.07) (Figure 2(h)).

### 3.4. HMGB1 Inhibitor Glycyrrhizin Attenuates the Effect of Diabetes

Western blot analysis was used to assess the effect of GA on diabetes-induced alterations of HMGB1, ERK1/2 activation, cleaved caspase-3, synaptophysin, TH, GS, and GLO1. Constant GA intake from the onset of diabetes significantly attenuated diabetes-induced upregulation of HMGB1, ERK1/2 activation, and cleaved caspase-3 by about 73%, 55%, and 78%, respectively (Figures 1(a), 1(b), and 1(c)). In the GA-fed diabetic rats, the decrease in synaptophysin, TH, GS, and GLO1 caused by diabetes was attenuated by about 58%, 55%, 79%, and 53%, respectively (Figures 1(d), 1(e), 1(f), and 1(g)). The levels of glutamate in the GA-treated diabetic rats (0.024 ± 0.002 nmol/*μ*L/*μ*g protein) were significantly less than those in the untreated diabetic rats (0.04 ± 0.015 nmol/*μ*L/*μ*g protein) (*P* = 0.045) (Figure 1(h)).

### 3.5. Retinal Expression of HMGB1 mRNA

**Figure 3:**
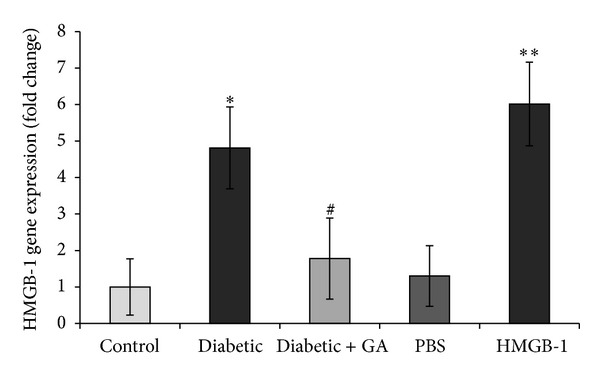
Gene expression of high-mobility group box-1 (HMGB1) in the retinas was quantified by RT-PCR using a primer given in the Materials and Methods section and was adjusted to the mRNA level of *β*-actin in each sample. Each measurement was performed at least 3 times. Results are expressed as mean ± SD of at least 6 rats in each group. GA: glycyrrhizic acid; PBS: phosphate buffer saline *P < 0.05 compared with nondiabetic controlled rats. **P < 0.05 compared with PBS. ^#^ *P* < 0.05 compared with diabetic rats.

The expression of HMGB1 mRNA in the retinas of diabetic rats was increased by about 4-fold compared to the retinas of nondiabetic rats. Intravitreal injection of HMGB1 resulted in increased HMGB1 mRNA expression by about 5-fold compared to the values obtained from the contralateral eye that received PBS alone. GA intake significantly attenuated diabetes-induced upregulation of HMGB1 mRNA by about 3.5-fold compared to untreated diabetic rats (Figure 3).

## 4. Discussion

In the present study, we investigated the pathological role of HMGB1 in diabetes-induced retinal neuropathy. Our previous studies showed that diabetes upregulates HMGB1 expression in the vitreous fluid and preretinal membranes from patients with PDR and its expression correlated with the activity of the disease and the levels of inflammatory biomarkers [20–22]. In addition, we demonstrated that HMGB1 expression was upregulated in the retinas of diabetic mice and rats [21, 23]. In the current study, we demonstrated that diabetes induced significant upregulation of cleaved caspase-3 and glutamate expression in the retinas of rats. On the other hand, diabetes induced significant downregulation of synaptophysin, TH, GS, and GLO1 in the retinas of rats. Furthermore, our data show that intravitreal injection of HMGB1 in normal rats mimics the effect of diabetes. The HMGB1 inhibitor GA attenuated diabetes-induced upregulation of HMGB1 protein and mRNA; cleaved caspase-3 and glutamate; and downregulation of synaptophysin, TH, GS, and GLO1 in the retinas of rats. Taken together, these findings suggest that HMGB1 contributes to retinal neuropathy induced by diabetes.

Diabetes induces retinal neurodegeneration as evidenced by the presence of apoptotic cells in all retinal layers [4, 25]. Activation of caspase-3 is part of the mechanism of apoptosis [25]. Several studies demonstrated that expression of active caspase-3, an indication of apoptosis, was upregulated in the diabetic retinas of human subjects [4] and rats [25]. In STZ-diabetic rat retinas, caspase-3 immunoreactivity was upregulated after 2, 8, and 16 weeks of diabetes [25]. However, Feit-Leichman et al. [26] demonstrated, in STZ-diabetic mice, upregulation of retinal caspase-3 activity at 1 month after induction of diabetes, which diminished to normal and began to increase again after approximately 6 months. They concluded that diabetes-induced degeneration of retinal capillaries can develop independent of neuronal loss. Synaptophysin is an integral protein of synaptic vesicles. It possibly serves multiple functions in synaptic vesicle formation and exocytosis, playing an important role in neurotransmitter delivery. It is widely used as one of the synaptic function markers and is also thought to be closely related to synaptogenesis and synaptic plasticity during neural tissue development. Synaptophysin knockout mice exhibited a significant decrease in synaptic vesicles in retinal rod photoreceptors which disturbs neurotransmitter release and synaptic network activity [27]. Previous studies demonstrated that 1 month of diabetes decreases retinal expression of synaptophysin [3, 28–30]. TH is the rate-limiting biosynthetic enzyme for dopamine synthesis. Therefore, the TH protein level is a marker of dopaminergic amacrine cells in the retina [1, 25, 31]. Several studies showed decreased TH protein levels in the diabetic retinas, reflecting reductions in the density of dopaminergic amacrine cells [1, 31–33]. The synaptically released glutamate is taken up by Müller cells where GS converts it into glutamine. Several studies found that the expression of GS was significantly decreased in the diabetic rat retinas [34, 35]. These dysfunctions resulted in elevated glutamate levels in the diabetic retinas [34, 36, 37], which might induce retinal neurodegeneration via glutamate excitotoxicity. In diabetes, there is accumulation of the AGEs precursor methylglyoxal (MG). In diabetic retinopathy, MG-derived AGEs are elevated in retina and are viewed to be causative in retinal injury and neurodegeneration [38–40]. Normally, MG is detoxified by the glyoxalase (GLO) enzyme system, composed of GLO1 and GLO2 [41]. A previous report demonstrated that GLO1 expression is reduced in the diabetic rat retinas [42]. It was also shown that GLO1 overexpression in diabetic rats prevents hyperglycemia-induced formation of MG-derived AGEs in the neural retina, prevents Müller glia dysfunction, and protects against capillary degenerative pathology [43].

In the present study, we demonstrated that, similar to diabetes, intravitreal injection of HMGB1 caused a significant upregulation of HMGB1 protein and mRNA and activated cleaved caspase-3 in the retina of normal rats. In addition, injection of HMGB1 tended to increase retinal glutamate expression, but this was not significant. In this regard, the recent finding that HMGB1 promotes glutamate release from gliosomes [19] suggests that its neurotoxicity is mediated, at least in part, by increased release of glutamate and enhanced excitotoxic neuronal death. On the other hand,* in vitro* studies demonstrated that glutamate can induce the release of HMGB1 from neuronal cells [14, 15]. Furthermore, intravitreal administration of HMGB1 induced downregulation of synaptophysin, TH, GS, and GLO1. These findings suggest that the early retinal neuropathy of diabetes involves the upregulated expression of HMGB1. Several studies showed that HMGB1 is massively released immediately after an ischemic insult and that it subsequently induces neuroinflammation in the postischemic brain [14, 15, 17]. To exert these activities, HMGB1 must transit from the nucleus, through the cytoplasm, to the outside of the cell. Several studies demonstrated that the inflammatory marker C-reactive protein [44] and the proinflammatory cytokines tumor necrosis factor-*α* (TNF-*α*) [45] and interleukin-1*β* (IL-1*β*) [46] induce translocation of HMGB1 from nucleus to the cytoplasm and its subsequent extracellular release from the cell.* In vitro* studies demonstrated that extracellular HMGB1 induced the proinflammatory biomarkers TNF-*α*, IL-1*β*, interferon-*γ*, intercellular adhesion molecule-1 (ICAM-1), inducible nitric oxide synthase (iNOS), and cyclooxgenase-2 in cultured neurons, astrocytes, microglia, and endothelial cells [14, 15, 17]. Moreover, cerebral microinjection of exogenous HMGB1 increased postischemic brain injury and upregulated the expression of iNOS and IL-1*β* [17]. These findings indicate that HMGB1 functions as a novel proinflammatory cytokine-like factor linking very early stages of cerebral ischemic injury with the activation of local neuroinflammatory response in the postischemic brain [14, 15]. Furthermore, it was also shown that extracellular HMGB1 released from the damaged brain neurons induces neuronal apoptosis and that this involves HMGB1-RAGE interaction [16]. In our laboratory, we recently demonstrated that diabetes induced significant upregulation of retinal expression of HMGB1, RAGE, activated NF-*κ*B, activated ERK1/2, and ICAM-1 and that intravitreal administration of HMGB1 in normal rats mimics the effect of diabetes [23]. We also showed that the HMGB1 inhibitor GA was effective in preventing diabetes-induced NF-*κ*B activation. In the present study, GA attenuated diabetes-induced ERK1/2 activation. In addition, coimmunoprecipitation studies showed that diabetes increases the interaction between HMGB1 and RAGE in the retina [23]. These findings suggest that activation of HMGB1/RAGE signaling axis with subsequent activation of NF-*κ*B and ERK1/2 is important in promoting diabetes-induced retinal neuropathy. Recently, it was reported that chronic neuroinflammation may be a driving force of progressive neurodegeneration and that HMGB1 provides the link between chronic neuroinflammation and progressive neurodegeneration in neurodegenerative diseases, such as Parkinson's disease [13].

To confirm the neuropathological implications of HMGB1 in diabetic retinopathy, the HMGB1 inhibitor GA was administered orally after inducing diabetes in rats. GA is known to bind directly to HMGB1 and inhibit its chemoattractant and mitogenic activities [24]. Interestingly, constant intake of GA significantly reduced retinal HMGB1 protein and mRNA expression induced by diabetes. In addition, GA attenuated diabetes-induced upregulation of cleaved caspase-3 and glutamate and counteracted the downregulation of synaptophysin, TH, GS, and GLO1 induced by diabetes without changing body weight or blood glucose levels. Our results are consistent with previous reports that demonstrated a neuroprotective effect of GA in animal models of brain ischemia [47], intracerebral hemorrhage [48], and spinal cord ischemia [49]. GA is known to possess glucocorticoid-like anti-inflammatory properties, due to its inhibitory activity on 11*β*-hydroxysteroid dehydrogenase [24]. Ohnishi et al. [48] demonstrated that the neuroprotective effect of GA on intracerebral hemorrhage-related pathogenic events was not mediated by glucocorticoid receptors or modulation of nitric oxide production and was reversed by exogenous HMGB1 application. These findings suggest that the protective effect of GA is mediated by its anti-HMGB1 activity and is irrelevant to the glucocorticoid system. It was demonstrated that the neuroprotective effect of GA was partly attributable to its inhibitory effect on HMGB1 release [47–49]. Furthermore, it was shown that GA affords protection in postischemic brain and spinal cord via its anti-inflammatory, antiapoptotic, antiexcitotoxic, and antioxidative effects [47, 49]. Together, these findings suggest that early retinal neuropathy induced by diabetes is, at least in part, attributable to diabetes-induced upregulation of HMGB1 expression and that inhibiting the release of HMGB1 with constant intake of GA results in less diabetes-induced retinal neuropathy. It is important to note that excessive intake of licorice may cause hypermineralocorticoidism-like syndrome characterized by sodium and water retention, potassium loss, edema, increased blood pressure, metabolic alkalosis, and depression of rennin-angiotensin-aldosterone system [50, 51].

In conclusion, our data point to a potential novel and pivotal role for HMGB1 as a mediator of diabetes-induced neuropathy in the retina. The HMGB1 inhibitor GA attenuated diabetes-induced upregulation of HMGB1 and diabetes-induced retinal neuropathy. Therefore, GA and other agents targeted to HMGB1 may provide novel therapeutic options for diabetic retinopathy.
